# Does education level affect the health status of the elderly? The chain mediating effect of internet use, health behavior and social class identity

**DOI:** 10.1371/journal.pone.0319389

**Published:** 2025-02-28

**Authors:** Ting Qin, Pingqiang Wei, Yuanyuan Xie

**Affiliations:** School of Literature and Journalism, Xihua University, Chengdu, Sichuan, China; University of Innsbruck: Universitat Innsbruck, AUSTRIA

## Abstract

**Background:**

China’s aging population is gradually increasing, and the health status of the elderly has become the focus of social attention. Education level is one of the important factors affecting the health status of the elderly. However, there are few studies on how education level specifically affects the health status of the elderly in China. Therefore, the purpose of this study is to explore the influence path of education level on the health status of the elderly in China, and to further study the mediating effect of Internet use, health behavior and social class identity in this process.

**Methods:**

This study is based on the latest version of the Chinese General Social Survey data as the basis of empirical analysis. Through multiple linear regression analysis, structural equation model analysis, Bootstrap method and robustness test, the relationship between education level, Internet use, health behavior, social class identity and the health status of the elderly is verified.

**Results:**

(1) Education level has a significant positive impact on the health status of the elderly in China (p < 0.05), which is, education level has a positive effect on the health status of the elderly in China. (2) Internet use, health behavior and social class identity have a significant mediating effect between education level and health status of Chinese elderly, and the mediating effect values are 0.024, 0.002 and 0.011, respectively. (3) Internet use, health behavior and social class identity play a chain mediating role in the impact of education level on the health status of the elderly, and the chain mediating effect value is 0.004.

**Conclusions:**

This study not only confirms the direct impact of education level on the health status of the elderly in China, but also reveals the indirect role of Internet use, healthy behavior and social class identity in this impact mechanism. These findings provide new perspectives and strategies for further improving the health status of the elderly in China.

## 1 Introduction

In China and even the world, the aging phenomenon is more and more serious, and the proportion of the elderly population is increasing year by year. This phenomenon has aroused widespread concern about the health status of the elderly. Aging not only indicates the fundamental change of social population structure, but also reveals the new dilemma of the elderly in terms of quality of life and health security. The assessment of health status is a multi-dimensional consideration, which covers the perfect state of physical, psychological and social adaptation [1]. However, under the unique cultural and socio-economic background of China, the health problems faced by the elderly are more complicated. The imperfection of the medical security system makes it difficult for the elderly in some areas to obtain high-quality and timely medical services [2]. Studies have shown that the higher the level of education of the elderly show stronger self-management and coping ability in the face of health challenges [3]. They can not only understand the doctor’s advice more accurately, make scientific diet and exercise plans, but also make use of various social resources more effectively, reducing the risk of illness. Nevertheless, the current research on the relationship between education level and the health status of the elderly is still scarce, especially in countries with unique social and cultural backgrounds such as China. Therefore, based on the existing research, we further explore the far-reaching impact of education on the health status of the elderly.

Education can not only improve knowledge and skills, but also shape individual lifestyles, cultivate healthy behavior habits, and form a positive psychological state. The level of education is not only a quantitative reflection of the time, level or academic qualifications of individuals receiving formal education, but also a key indicator to reveal the position of individuals in the knowledge system and measure the overall progress of education [4]. Importantly, the level of education is closely related to the individual’s cognitive ability, information processing ability and social adaptability, which together constitute the basis for the effective operation of the individual in society. At the same time, individuals with higher education are more likely to choose a reasonable diet and maintain regular exercise. These habits are crucial individuals to maintain the long-term health. The elderly with higher education have obvious advantages in physical health, mental health and social function. The study found that the elderly with higher education have a significantly lower risk of chronic diseases, and a slower rate of cognitive decline. They are more willing and able to actively participate in social activities and enjoy a higher quality of life [5,6]. This suggests that education may have a profound positive impact on the health of the elderly by improving the level of individual health knowledge, strengthening self-management ability and enriching social interaction. Based on this, this study proposes hypothesis 1: education level positively affects the health status of the elderly.

With the development of Internet technology, our way of life, means of communication and access to information have undergone earth-shaking changes, which has become an important part of life. For the elderly, Internet technology provides them with more convenient access to information, and plays a bridge role between social communication and leisure and entertainment [7–9]. The elderly can quickly consult professional doctors through online medical services, and timely understand their own health status, so as to facilitate early detection, early prevention and early treatment of physical diseases [10,11]. Health knowledge on the Internet not only scientifically guide the diet and exercise of the elderly, but also prevent and control the occurrence of chronic diseases. Studies have shown that there is a positive correlation between the use of the Internet and the health status of the elderly [12–15]. The elderly who actively use the Internet show better performance in cognitive ability, self-care and participation in social activities [16]. Based on this, this study proposes hypothesis 2: Internet use plays a mediating role in the impact of education level on the health status of the elderly.

Health behavior refers to the behavior patterns that help to maintain and promote individual health. At the same time, regular exercise and reasonable eating habits are equally important for mental health, which can relieve stress and improve the quality of life [17]. Healthy behavior is very important for health status of the elderly [18,19]. With the increase of age, their own metabolic function is weakened, immunity is reduced, and the elderly are prone to various diseases. By adhering to a healthy lifestyle, the elderly can improve their health, delay the aging process, and maintain a high quality of life [20]. Those who regularly exercise and maintain a balanced diet, as well as those who quit smoking and limit alcohol, have better health [21]. The higher the level of education of the elderly are more willing to adopt health behavior advice. The elderly with higher education of are more willing to adopt health behavior advice, and they pay more attention to self-care and have stronger health awareness [22,23]. Based on this, this study proposes hypothesis 3: health behavior plays an intermediary role in the impact of education level on the health status of the elderly.

Social class identity is the individual’s perception and identity of their position in the social hierarchy [24]. Social class identity has a significant impact on individual mental health and physical health, especially in the elderly [25,26]. The elderly with high social class identity are more likely to seek and accept health information, and to adopt a healthy lifestyle. Studies have shown that older people who identify themselves as belonging to a higher social class have better self-rated health status than other older people [27]. The elderly with higher social class identity are more inclined to actively seek medical help when facing health problems, improving the cure rate and quality of life [28]. Older people with higher levels of education generally have better career opportunities and social status, directly enhancing their social class identity. This higher social class identity translates into a healthier lifestyle and a more positive state of health. Based on this, this study proposes hypothesis 4: Social class identity plays a mediating role in the impact of education level on the health status of the elderly.

There is a close relationship between Internet use, healthy behavior and social class identity. The three factors interact with each other and affect the health status of the elderly. Internet use provides elderly people with convenient access to information, enabling them to obtain health knowledge and healthy lifestyle information on the Internet [29,30]. Internet use can make the elderly more widely involved in social life, communicate with different classes of people, and easily enhance or change their social class identity [31]. Such class identity can also affect the psychological state and healthy behavior of the elderly [32,33]. The elderly with higher social class identity are willing to choose positive health behaviors to prevent and control the occurrence of chronic diseases, thus their quality of life and health status can be improved. Based on this, this study proposes hypothesis 5: Internet use, health behavior and social class identity play a chain mediating role in the impact of education level on the health status of the elderly.

## 2 Materials and methods

### 2.1 Data sources

This study is based on the latest 2021 data released by China general social survey (CGSS (2021)). The purpose of CGSS (2021) data is to systematically monitor the interaction and change of social structure and quality of life through the annual social survey of urban and rural families, collect and establish a tracking data database of social change trends, and provide research resources for domestic and international academic circles. The overall issue framework of the data is the social structure, quality of life and the internal connection mechanism between them. The data collection work is carried out by the China Survey and Data Center of Renmin University of China. The data research has high quality, authoritative, extensive and continuous characteristics. The studies involving humans were approved by the Ethics Committee of Renmin University of China and they were conducted in accordance with the local legislation and institutional requirements. The oral consent of the participants to participate in this study was obtained. The initial sample size of the data is 8148, including 19 provinces, autonomous regions and municipalities in China. Based on the research needs, we have done the sample screening work: Firstly, eliminate samples under the age of 60. Secondly, eliminate the missing values or unqualified samples in the variables. Finally, get 2929 valid sample data. The sample collected important indicators such as education level, health status, Internet use, healthy behavior, social class identity, and sociodemographic characteristics (such as gender, age, ethnicity, religion, education, occupation, income, property rights, wealth, etc.) of the elderly, which can meet the needs of research.

### 2.2 Variables measurement

#### 2.2.1 Dependent variable.

Health status mainly includes physical health status and mental health status. This study refers to Hou and Zhang [34], Zeng and Yang [35], Liu et al., on the health status assignment method [36], and selects the problem in CGSS (2021) “In general, what do you think of your health status (here health includes physical and mental health)?” as a variable of health status. A total of 5 points, 1 = poor, 5 = very good, the larger the value, the better the health status.

#### 2.2.2 Independent variable.

Choose “What is your current highest level of education?” as an independent variable. A total of 5 points, 1 = without any education, 5 = postgraduate and above, the greater the value, the higher the level of education.

#### 2.2.3 Mediator variable.

Choose “over the past year, your use of the Internet (including mobile Internet)?” as a mediating variable. A total of 5 points, 1 = never, 5 = very frequently, the greater the value, the higher the frequency of Internet use.

Choose “Do you agree or disagree with the statement that people have serious health problems because they behave in a way that impairs their own health (e.g., smoking, taking drugs)?” as a mediating variable. A total of 5 points, 1 = very disagree, 5 = very agree, the larger the value, the better the health behavior.

Social class identity is the subjective perception of residents on their own social level [37], which has been paid more and more attention in the study of the health status of the elderly. This study refers to the classification method used by Liang and Wang [38], Mi et al. [39], combined with the actual situation in China, according to the 10-level scale to divide the social class. Select “Generally speaking, in the current society, which level of society are you in?” as a mediating variable. A total of 10 points, 1 = the bottom, 10 = the top, the greater the value, the higher the social class identity.

#### 2.2.4 Control variable.

Gender, ethnicity, household registration, religious belief, marital status, social justice cognition, subjective well-being and other socio-demographic characteristics were selected as control variables.

### 2.3 Analysis method

#### 2.3.1 Multiple linear regression model.

The multiple linear regression model is shown in formula (1):


$$\text{Y}={\text{β}}_{0}+{\text{β}}_{1}{\text{X}}_{1}+{\text{β}}_{2}{\text{X}}_{2}+{\text{β}}_{3}{\text{X}}_{3}+{\text{β}}_{4}{\text{X}}_{4}+{\text{β}}_{5}{\text{X}}_{5}+\epsilon$$ (1)

In formula (1), Y represents the score of the health status of the elderly, ${\text{X}}_{1}$ represents the level of education, ${\text{X}}_{2}$ represents the socio-demographic characteristics, ${\text{X}}_{3}$ represents the use of the Internet, ${\text{X}}_{4}$ represents healthy behavior, and ${\text{X}}_{5}$ represents the social class identity. ${\text{β}}_{0}$ is the intercept term, indicating the health status reference value predicted by the model when all independent variables are zero. ${\text{β}}_{1}$–${\text{β}}_{5}$ are regression coefficients, which represent the influence strength of each variable on the dependent variable Y respectively. Among them, ${\text{β}}_{1}$ is the focus of this study, indicating the direct impact of education level on women ‘s health status. *ϵ* is the error term, which represents the influence of other factors that are not observed in the model on health status.

#### 2.3.2 Structural equation model.

The structural equation is constructed as shown in formula (2):


Y=By+Γx+ζ (2)

In formula (2), y is the endogenous variable vector, x is the exogenous variable vector, B is the path coefficient matrix between endogenous variables, Γ is the path coefficient matrix between exogenous variables and endogenous variables, and ζ is the residual term of the structural equation model. Then, through the constructed structural equation model, the Bootstrap method is used to estimate the mediating effect and to test the significant effect of the chain mediating effect.

## 3 Results

### 3.1 Descriptive statistical results

This study mainly discusses the relationship between education level and the health status of the elderly. According to the descriptive results of each variable in Table 1, the sample data of health status (dependent variable) is 2929, the score range of health status is from 1 to 5, and the mean value is 3.123 (standard deviation SD = 0.696), indicating that most of the samples are in good health. The mean value of education level (the main independent variable) is 1.849 (standard deviation SD = 0.565), the minimum value is 1 (“no education”), and the maximum value is 5 (“postgraduate education”), which shows that the education level of the elderly in China is generally not high. The descriptive statistical results of each variable are shown in Table 1.

**Table 1:** Descriptive result statistics of each variable.

Variable	N	Minimum value	Maximum value	Mean value	Standard deviation
Social justice recognition	2929	0.000	1.000	0.650	0.477
Subjective well-being	2929	0.000	1.000	0.568	0.495
Education degree	2929	1.000	5.000	1.849	0.565
Internet use	2929	1.000	5.000	2.083	1.532
Healthy behavior styles	2929	1.000	5.000	3.155	0.638
Class identity	2929	1.000	10.000	4.328	1.984
Health conditions	2929	1.000	5.000	3.123	0.696

**Fig 1:**
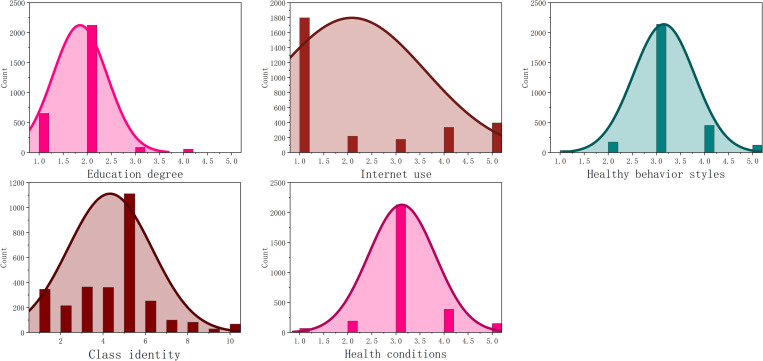
Histogram of normality test.

Before the formal analysis, this study conducted a normality test on the five variables of education level, Internet use, health behavior, social class identity, and health status to ensure the validity of the subsequent analysis and the reliability of the results. This study uses IBM SPSS Statistics 26 software for normality test, and the normality test diagram is shown in Fig 1.

According to Fig 1, it can be seen that the variable is basically acceptable normal distribution, and the next research and analysis can be carried out.

### 3.2 Multiple regression results

This study takes the health status of the elderly in China as the dependent variable, the level of education as the independent variable, the use of the Internet, healthy behavior, and social class identity as the control variables, and selects social equity cognition, marital status, subjective well-being, and other socio-demographic characteristics as control variables to establish a multiple linear regression model. The construction and results of this model are shown in Table 2. In the model 1, only socio-demographic characteristics were included as control variables, including gender, ethnicity, household registration, religious beliefs, marital status, social equity cognition, and subjective well-being; in model 2, the Internet usage factor is added on the basis of model 1. In model 3, health behavior factors were added on the basis of model 2. In model 4, on the basis of model 3, the social class identity factor is added; in model 5, all independent variables, mediating variables and control variables are integrated.

**Table 2:** Multiple linear regression results of influencing factors of health status.

Variable	Model 1	Model 2	Model 3	Model 4	Model 5
Constant term	3.377** (0.067)	3.455** (0.070)	3.547** (0.097)	3.685** (0.100)	3.684** (0.108)
Internet use	—	0.030** (0.009)	0.029** (0.009)	0.028** (0.009)	0.029** (0.009)
Healthy behavior styles	—	—	0.028 (0.021)	0.026 (0.020)	0.026** (0.020)
Class identity	—	—	—	0.034** (0.006)	0.034** (0.007)
Education degree	—	—	—	—	0.041** (0.025)
N	2929	2929	2929	2929	2929
Adjusted $R^{2}$	0.043	0.047	0.048	0.055	0.056

Note: *p < 0.05, **p < 0.01, standard deviation in parentheses.

The results show that in model 5, after the education level is included in the model, the regression coefficient is 0.041** (p = 0.000 < 0.01), and the results significantly positively affect the health status. This result shows that the hypothesis 1 of this study is established, which means the education level has a positive impact on the health status of the elderly. By comparing the adjusted R square values of Model 1 to Model 5, it can be seen that it gradually increases with the increase of variables. The adjusted R square value of Model 5 is 0.056, which is significantly improved compared with the 0.043 of Model 1.This shows that with the increase of variables, the model’s explanation of health status is more comprehensive and the explanatory power is enhanced. According to the results of Model 5, under the influence of control variables, education level has a positive impact on the health status of the elderly in China, and the health status is improved by 0.013 points on average, meaning the higher the education level is, the more positive the health status of the elderly is.

According to Table 2, the regression coefficient of Internet use is 0.030 (p = 0.001 < 0.01), the regression coefficient of healthy behavior is 0.028 (p = 0.000 < 0.01), and the regression coefficient of social class identity is 0.034 (p = 0.000 < 0.01). It shows that Internet use, health behavior and social class identity have a positive impact on the health status of the elderly, indicating that these three variables are important factors affecting the health status of the elderly.

In summary, through multiple regression analysis, it is proved that education level has a significant positive impact on the health status of the elderly. After adding the mediating variables of Internet use, health behavior and social class identity, it still shows that education level has a positive impact on the health status of the elderly. In the follow-up part, this study will use the structural equation model to further explore the results.

### 3.3 Structural equation model results

According to the social demographic characteristics, education level, Internet use, health behavior, social class identity and health status, the structural equation model of this study was established. The structural equation model parameters were calculated as shown in Table 3.

**Table 3:** Model fitting degree table.

Index	Fit index	Standard	Actual value	Result
Absolute index	χ2/df	<3	0.900	Excellent
	GFI	>0.9	1.000	Excellent
	RMR	<0.05	0.000	Excellent
Value-added indicators	RMSEA	<0.10	0.091	Excellent
	CFI	>0.9	0.988	Excellent
	NFI	>0.9	1.000	Excellent
	NNFI	>0.9	1.025	Excellent

It can be seen from Table 3 that the values fitted by the above models are within the standard range, indicating that the fitting degree of this model is very good, and the data of this study has a good fitting effect. Therefore, the results of this model are shown in Table 4.

**Table 4:** Results of structural equation model.

Latent variable	→	Manifest variables	Standardized coefficient	Significant P value
Education degree	→	Internet use	0.573	0.000
Education degree	→	Healthy behavior styles	0.098	0.009
Education degree	→	Class identity	0.090	0.000
Education degree	→	Health conditions	0.622	0.002
Internet use	→	Healthy behavior styles	0.183	0.014
Internet use	→	Class identity	0.130	0.009
Internet use	→	Health conditions	0.222	0.009
Healthy behavior styles	→	Class identity	0.010	0.004
Healthy behavior styles	→	Health conditions	0.117	0.005
Class identity	→	Health conditions	0.381	0.000

Note: → denotes regression influence relation or measurement relation.

**Fig 2:**
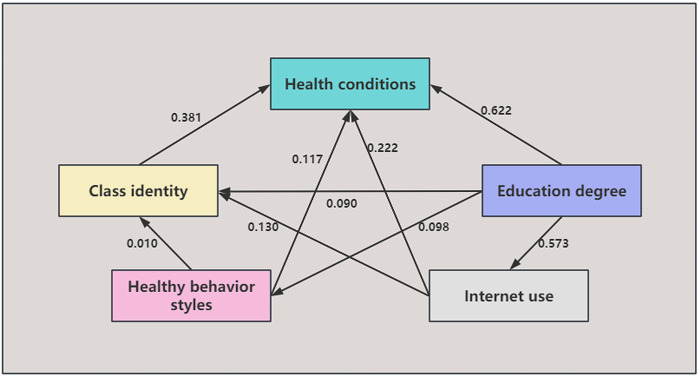
Path result diagram of structural equation model.

It can be seen from Table 4 that the measured variables of all factors are P < 0.05, indicating that these variables are statistically significant. The path of this model is from the latent variable to explicit variable: “education level→Internet use, education level→healthy behavior pattern, education level→social class identity, education level→health status, Internet use→healthy behavior pattern, Internet use→social class identity, Internet use→health status, healthy behavior pattern→social class identity, healthy behavior pattern→health status, social class identity→health status”, all paired items P < 0.05, indicating that all paths are significant, so all paths established in this model are effective. The influence coefficients of each path are: 0.573, 0.098, 0.090, 0.622, 0.183, 0.130, 0.222, 0.010, 0.117, 0.381, indicating that these paths play a significant role in the model. In particular, the influence coefficient of education level on health status (0.622) is the largest, indicating that education level plays an important role in the health status of the elderly. The path diagram of the structural equation model is shown in Fig 2.

### 3.4 Mediation model results

In this study, Bootstrap method was used to test the mediating effect. Taking education level as an independent variable, health status as a dependent variable, Internet use, health behavior and social class identity as mediating variables, a mediating model was established. The results of Bootstrap analysis of mediating effect are shown in Table 5.

**Table 5:** Bootstrap analysis of mediating effect.

Category	Effect	Boot SE	BootLLCI	BootULCI
Educational level→Internet use→Health status	0.024	0.006	0.005	0.034
Education level→Health behavior→Health status	0.002	0.001	0.001	0.003
Education level→Social class identity→Health status	0.011	0.003	0.003	0.015
Education level→Internet use→Health behavior→Health status	0.001	0.001	0.001	0.002
Education level→Internet use→Social class identity→Health status	0.001	0.001	0.001	0.004
Education level→Health behavior→Social class identity→Health status	0.002	0.003	0.001	0.007
Education level→Internet use→Health behavior→Social class identity→Health status	0.004	0.002	0.003	0.010

Note: BootLLCI refers to the lower limit of 95% interval of Bootstrap sampling, and BootULCI refers to the upper limit of 95% interval of Bootstrap sampling.

Education level not only directly affects health status, but also indirectly affects health status through Internet use, health behavior and social class identity. As shown in Table 5, the mediating effect of education level on Internet use is 0.024, and the 95% confidence interval (CI) is [0.001, 0.004]. The path interval does not include the number 0, indicating that this mediating effect path exists. It shows that education level can have a significant indirect positive impact on health status through Internet use, which supports hypothesis 2 of this study. The mediating effect of education level on health behavior is 0.002, and the 95% confidence interval (CI) is [0.001, 0.003]. The path interval does not include the number 0, indicating the existence of this mediating effect path. It shows that education level can have a significant indirect positive impact on health status through Internet use, which supports hypothesis 3 of this study. The mediating effect of education level on social class identity is 0.011, and the 95% confidence interval (CI) is [0.003, 0.015]. The path interval does not include the number 0, indicating that this mediating effect path exists. It shows that education level can have a significant indirect positive impact on health status through social class identity, which supports hypothesis 4 of this study.

The chain mediating effect path is analyzed. According to the mediating path of “education level→Internet use→healthy behavior→health status”, the mediating effect is 0.001, and the 95% interval does not include the number 0 [0.001, 0.002], indicating that this mediating effect path exists. For the mediating path of “education level→Internet use→social class identity→health status”, the mediating effect is 0.001, and the 95% interval does not include the number 0 [0.001, 0.004]. Showing this mediating effect path exists. According to the mediating path of “education level→healthy behavior→social class identity→health status”, the mediating effect is 0.002, and the 95% interval does not include the number 0 [0.001, 0.007], indicating the existence of this mediating effect path. According to the mediating path of “education level→Internet use→healthy behavior→social class identity→health status”, the mediating effect is 0.004, and the 95% interval does not include the number 0 [0.003, 0.010].Therefore, this mediating effect path exists. The existence of four chain mediating effect paths shows that Internet use, health behavior and social class identity play a chain mediating role in the influence of education level on the health status of the elderly, which supports hypothesis 5 of this study.

### 3.5 Robustness test results

Due to the variables of the model study, there may be unobserved influencing factors. For example, variables such as education level and Internet use may be affected by factors such as income and regional differences. Therefore, in order to test the conclusion of the model, this study further tests the robustness of the model by adding the two variables of residents’ income and provincial and municipal areas.

Through the multiple linear regression model, the two variables of residents’ income and provincial and municipal areas are added. The selection of these two variables is of great significance. Residents’ income is closely related to the quality of life and the ability to obtain medical resources of the elderly, which may indirectly affect their health behavior and health status, and may also be related to their education level. Provinces and cities cover the differences in regional economic development level, distribution of medical facilities, social and cultural environment, etc. These factors have a potential impact on the health status of the elderly and Internet use at the macro level. The robustness test results of this study are shown in Table 6.

**Table 6:** Robustness test results.

Variable	Model 1	Model 2	Model 3	Model 4	Model 5
Constant term	3.375** (0.067)	3.453** (0.070)	3.544** (0.094)	3.683** (0.098)	3.686** (0.104)
Internet use	—	0.030** (0.009)	0.032** (0.009)	0.034** (0.007)	0.035** (0.008)
Healthy behavior styles	—	—	0.029** (0.022)	0.031** (0.020)	0.033** (0.021)
Class identity	—	—	—	0.034** (0.007)	0.035** (0.007)
Education degree	—	—	—	—	0.039** (0.025)
N	2929	2929	2929	2929	2929
Adjusted $R^{2}$	0.041	0.042	0.044	0.048	0.057

Note: *p < 0.05, **p < 0.01, standard deviation in parentheses.

The results of Table 6 show that the key variables of this research model remain significant after adding variables of residents’ income, provinces and municipalities. Internet use variables have a significant positive impact on health status in each model. The influence coefficients of Internet use variables are $\beta_{model2}^{Internetusage}=0.030$, $\beta_{model3}^{Internetusage}=0.032$, $\beta_{model4}^{Internetusage}=0.034$, $\beta_{model5}^{Internetusage}=0.035$, respectively, indicating that the role of the Internet in the health promotion of the elderly is relatively stable and is not influenced by new variables. The influence coefficients of health behavior variables are $\beta_{model3}^{Healthybehavior}=0.029$, $\beta_{model4}^{Healthybehavior}=0.031$, $\beta_{model5}^{Healthybehavior}=0.033$, respectively, indicating that the positive impact of health behavior on health status is robust. The variable of social class identity continues to be significant in the model. The influence coefficients of the variable of social class identity are $\beta_{model4}^{Socialhierarchy}=0.034$, $\beta_{model5}^{Socialhierarchy}=0.035$ respectively, which still significantly affects the health status and further proves its stable role in the model. The influence coefficient of education level in model 5 is $\beta_{model5}^{Educationallevel}=0.039$. Although the coefficient of the original model has changed, it still has a positive impact on health status, which means that after considering residents ‘income and regional differences, the positive effect of education level on the health of the elderly still exists, and the overall structure of the model in this study has not changed fundamentally.

From the perspective of the explanatory power of the model, the adjusted ${\text{R}}^{2}$ value shows an upward trend with the increase of variables, from 0.041 in model 1 to 0.057 in model 5, indicating that the addition of new variables enhances the explanatory power of the model on the health status of the elderly, so that the model can more fully capture the factors affecting health and further support the robustness of the model.

In summary, through the robustness test, it can be seen that the core conclusions of this study are still valid after considering the potential interference factors, showing the positive impact of education level on the health status of the elderly and the role of various mediating variables have strong robustness, which provides a strong support for the reliability of the research results.

## 4 Discussion

### 4.1 Education level positively affects the health status of the elderly

The results show that education level has a positive impact on the health status of the elderly, which means education level positively affects the health status of the elderly, which verifies hypothesis 1 and is consistent with the existing research results [40–42]. The elderly with high education level have stronger coping ability in psychological adjustment [43,44]. They have rich psychological resources and diversified coping strategies, and can flexibly cope with various setbacks and pressures in life. Whether they are faced with major life events such as retirement, widowhood, or small problems in daily life, they can face them with a positive attitude, thereby reducing the potential threat of negative emotions to physical health. The level of education also has a profound impact on the social participation and social activities of the elderly. Highly educated seniors tend to be able to build broader and deeper social networks and engage in more social activities [45]. This not only helps them maintain close ties with society, reduce loneliness and depression, but also improves the overall quality of life by increasing physical activity and social interaction. Highly educated elderly people also show obvious advantages in the use and integration of medical resources. They have higher medical literacy, can accurately understand and accept the doctor’s advice, and develop more accurate and effective treatment and rehabilitation programs. At the same time, they are also good at using modern information technology means, such as Internet medical platform, to obtain cutting-edge medical information and professional health consultation [46,47]. This allows them to make more scientific decisions in the face of health problems, so as to protect their own health.

### 4.2 Internet use plays an intermediary role in the influence of education level on the health status of the elderly

The results show that there is a significant positive correlation between education level, Internet use and the health status of the elderly. Internet use plays an intermediary role in the influence of education level on the health status of the elderly, which verifies the research hypothesis 2 and is consistent with the existing research results [48]. Education level is an important factor in predicting the health status of the elderly. However, the impact of education level on health status is not direct and single, but the result of a combination of multiple pathways and mechanisms [49,50]. More educated older people are more likely to use the Internet, where they can access health information, manage their personal health, and share health experiences with other older people [51,52]. According to the research, among the elderly with higher education, the group who often use the Internet is significantly better than the group who do not use the Internet in terms of health knowledge score, self-management ability and overall health status [53]. In addition, the Internet is not only a platform for information acquisition, but also a bridge between people. For the elderly, tools such as social media and online forums not only break the limitations of geography and time, but also provide them with a space to communicate, share and build social networks with others. This social interaction not only helps to alleviate the loneliness of the elderly, but also virtually improves their mental health [54]. It is worth mentioning that highly educated elderly people have obvious advantages in this regard, and they are more able to make full use of these tools to build a closer and healthier social circle.

### 4.3 Health behavior plays an intermediary role in the influence of education level on the health status of the elderly

The results show that there is a significant positive correlation between education level, health behavior and health status of the elderly. Health behavior plays an intermediary role in the influence of education level on the health status of the elderly, which verifies the research hypothesis 3. The higher the level of education of the elderly pay more attention to self-care and health knowledge acquisition. They are more willing to take the initiative to accept the scientific concept of health, and are more willing to adopt positive health behavior [55]. This willingness is mainly reflected in daily diet and exercise habits, as well as early physical examination and disease prevention, which directly promote their physical and mental health [56,57]. On the contrary, the lower the education level of the elderly may lack relevant health knowledge and awareness, still adhere to smoking, long-term drinking, etc., less to take health behavior advice, thus increasing the risk of disease [58–60]. In addition, the elderly with higher education level are better at using medical resources, and they will take the initiative to seek medical advice, understand health knowledge and preventive measures [22]. This positive health behavior is conducive to timely detection of health problems in the elderly, and can be more effectively treated after the disease occurs.

### 4.4 Social class identity plays an intermediary role in the influence of education level on the health status of the elderly

The results show that there is a significant positive correlation between education level, social class identity and the health status of the elderly. Social class identity plays an intermediary role in the influence of education level on the health status of the elderly, which verifies the research hypothesis 4. The level of education not only directly affects the individual’s knowledge reserve and cognitive ability, but also shapes the individual’s social status perception and self-worth assessment at a deep level. This kind of deep perception and evaluation is the recognition of social class, which has a significant impact on the psychological state and lifestyle of the elderly [61]. The higher the education level of the elderly tend to have a higher social class identity. This sense of identity not only enhances their self-esteem and self-confidence, but also encourages them to focus more on health management and disease prevention [62]. The more educated the elderly are, the stronger their sense of social class identity is, and they are more inclined to adopt healthy lifestyles, such as balanced a diet, moderate exercise and regular work and rest [63]. The higher the education level of the elderly the higher their knowledge reserve and cognitive ability is, and they can quickly understand and receive health information, and ultimately choose healthy behavior. These healthy behaviors are maintained through social class identity [64]. This shows that the higher the education level of the elderly, the higher the social class identity, which further encourages them to choose healthy behavior, which in turn has a positive impact on their health status.

### 4.5 Internet use, health behavior and social class identity play a chain mediating role in the influence of education level on the health status of the elderly

The results show that Internet use, health behavior and social class identity play a chain mediating role in the influence of education level on the health status of the elderly, which verifies the research hypothesis 5. The more educated the elderly are, the more willing they are to choose to use the Internet to acquire and learn health knowledge through network technology [65,66]. Mastering and applying this health knowledge is conducive to the formation of healthy behaviors for the elderly. At the same time, through the Internet platform, the elderly participate in a wider range of social activities and interact with people of different classes, which can not only locate their own social class identity, but also help to enhance their social class identity and have a positive impact on their mental state and health status. The elderly with higher education level use the Internet more frequently than those with lower education level [67,68]. Therefore, we conclude that the higher the education level of the elderly through the high frequency of the use of the Internet, may get more health information, health behavior is more scientific. In social interaction, this part of the elderly is also more confident and stronger sense of belonging, which is conducive to their social class identity and health status. Internet use has a significant effect on improving the health behavior of the elderly [69,70]. The elderly who use the Internet frequently may have higher self-discipline and scientificity in health habits. This kind of healthy behavior directly improves the physical health of the elderly. At the same time, it enhances the recognition of social class and indirectly promotes the improvement of mental health level.

### 4.6 The potential impact of the post-COVID-19 epidemic on the health status of the elderly

In 2021, during the COVID-19 epidemic, the social activities of the elderly are limited, and mental health problems are highlighted. At the same time, the epidemic has also exacerbated the unequal distribution of medical resources, making it more difficult for elderly people with low education levels to access quality medical services [71,72]. These factors may have an impact on the relationship between education and health status. In addition, the epidemic has also had a profound impact on the lifestyle of the elderly. The elderly have less exercise and their eating habits may change, which may have a negative impact on their health status [73,74]. Therefore, in the post-COVID-19 epidemic period, we need to pay more attention to the health status of the elderly and take positive measures to reduce the impact of the epidemic on them.

## 5 Conclusion

By exploring the impact of education level on the health status of the elderly in China, and the mediating role of Internet use, healthy behavior and social class identity, this study draws the following conclusions:

1. Education level positively affects the health status of the elderly. The higher the education level of the elderly the more willing the elderly are to pay attention to personal health, and they will take the initiative to acquire health knowledge, and actively develop scientific health behavior habits. When facing health problems, they are more willing to take active treatment and rehabilitation measures to maintain their health status.
2. Internet use, health behavior and social class identity have a significant mediating effect between education level and health status of the elderly. The elderly with higher education level are more willing to learn health knowledge through the Internet, improve their health literacy, and form good health behaviors that have a positive impact on their health status. At the same time, the elderly with higher education tend to have a higher social class identity, improving their self-esteem and self-confidence and making them more willing to pay attention to their health status.
3. Internet use, health behavior and social class identity play a chain mediating role in the impact of education level on the health status of the elderly. These three factors including Internet use, healthy behavior and social class identity interact each other. The higher the education level of the elderly, more willing to get health information through the use of the Internet, in order to form a scientific way of health behavior, and then actively affect their social class identity, and ultimately promote their health status.

In summary, the conclusions of this study are conducive to our deeper understanding of the influencing factors of the health status of the elderly, and the formulation of accurate health policies for the elderly by government departments. At the same time, it provides new research perspectives for researchers to study social security system and public health policy.

## 6 Limitations of the study and future directions

This study has made some achievements in exploring the relationship between education level, Internet use, health behavior, social class identity and the health status of the elderly in China, but there are still limitations. First, the timeliness of data lags behind. We use the latest version of China’s comprehensive social survey data (2021), but society, Internet technology, and the habits of the elderly are constantly changing, and the survey data cannot fully reflect the current social situation. Future research can track the latest data on the basis of the Chinese General Social Survey questionnaire, so as to capture the latest issues of Internet use and health behavior of the elderly. Second, there may be subjectivity or inaccuracy in defining and quantifying variables such as Internet use, healthy behavior, and social class identity. Future research can develop more accurate and objective measurement tools to reduce subjective bias. Third, there may be endogenous problems. Factors such as education level, Internet use, and healthy behavior may also be affected by other unobserved variables. Future research can use panel data, experimental design and other methods to better deal with endogenous problems.
